# Mass Coral Bleaching in 2010 in the Southern Caribbean

**DOI:** 10.1371/journal.pone.0083829

**Published:** 2014-01-06

**Authors:** Jahson Berhane Alemu I, Ysharda Clement

**Affiliations:** 1 Biodiversity and Ecology Research Programme, Institute of Marine Affairs, Hilltop Lane, Chaguaramas, Trinidad and Tobago; 2 Department of Life Sciences, University of the West Indies, Saint Augustine Campus, Saint Augustine, Trinidad and Tobago; University of Aveiro, Portugal

## Abstract

Ocean temperatures are increasing globally and the Caribbean is no exception. An extreme ocean warming event in 2010 placed Tobago's coral reefs under severe stress resulting in widespread coral bleaching and threatening the livelihoods that rely on them. The bleaching response of four reef building taxa was monitored over a six month period across three major reefs systems in Tobago. By identifying taxa resilient to bleaching we propose to assist local coral reef managers in the decision making process to cope with mass bleaching events. The bleaching signal (length of exposure to high ocean temperatures) varied widely between the Atlantic and Caribbean reefs, but regardless of this variation most taxa bleached. *Colpophyllia natans*, *Montastraea faveolata* and *Siderastrea siderea* were considered the most bleaching vulnerable taxa. Interestingly, reefs with the highest coral cover showed the greatest decline reef building taxa, and conversely, reefs with the lowest coral cover showed the most bleaching but lowest change in coral cover with little algal overgrowth post-bleaching.

## Introduction

Mass coral bleaching is one of the major threats to coral reef ecosystems [1], [2] exacerbating coral reef decline in the Caribbean region [3]. Often mass coral bleaching events are as a result of the prolonged exposure of corals to unusually warm ocean temperatures, resulting in the expulsion of symbiotic algae from host corals. However, not all coral taxa are equally susceptible to bleaching [4]. Some taxa may bleach, whereas others exposed to the same heat stress may not bleach or show intermediate signs of bleaching.

Tobago's coral reefs are located at the southern extreme of the Caribbean, exposed regularly to the rich outflow of the South American mainland [5], [6] and represent some of the most understudied reefs in the region [6]. In 2005, a mass bleaching event resulted in up to 85% bleaching and up to 75% mortality of important reef-building species such as *Colpophyllia natans* (Houttuyn 1772) and *Diploria* spp. in Tobago [7], [8]. Post bleaching the result was an outbreak of coral disease and macroalgae, and ultimately reduced reef quality.

Still recovering from the 2005 mass bleaching event, Tobago was again affected by another mass bleaching event in 2010. This study sought to present a quantitative analysis of the effects of mass coral bleaching on four main reef building taxa at three popular reefs in Tobago, West Indies. In this study, bleaching response was considered to be either bleaching or mortality in response to thermal stress by corals. Ultimately, our goal is to identify nodes of reef resilience on Tobago.

### Site description

**Figure 1:**
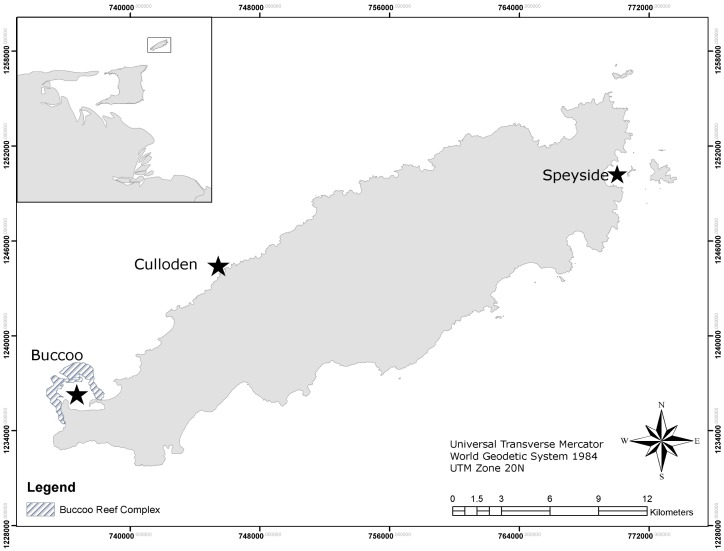
Map of Tobago showing the locations of sampling sites (black stars) with inset of Trinidad and Tobago.

The study was conducted on Buccoo Reef (11°11′N, 60°49′W), Culloden Reef (11°14′N, 60°45′W) and Speyside (11°17′N, 60°30′W). These represent three of the largest reefs systems around Tobago (Figure 1). Buccoo and Culloden Reefs are old coralline reefs, whereas Speyside Reefs are coral communities established on old igneous rock. The Buccoo Reef is a composite of five emergent reef flats that slope gradually to depths of about 30 m and fringes a shallow lagoon, seagrass beds and a mangrove wetland [9]. Mean coral cover on Buccoo Reef was 25.2%. Culloden Reef begins about 100 m offshore and has a horse-shoe shape, where the reef slopes gently to depths below 25 m. In the bay area, the reef slopes steeply to about 12 m [9] with many large sand channels in the deep area of the fore reef perpendicular to the shore. These channels are separated by reef buttresses composed of stony corals [9]. Mean coral cover at Culloden was 26.3%. Speyside Reef is a fringing reef dominated by stony corals and sponges. The reef extends to depths below 35 m [9]. Strong, year-round currents characterize this site as a result of the convergence of the Atlantic Ocean and Caribbean Sea, and open exposure to the North East Trade Winds. Mean coral cover at Speyside is 17.6%. All surveys were conducted at the same depth (10–12 m) an area used in long term reef monitoring. Dominant hard coral taxa on all study areas were *C. natans* (Houttuyn, 1772), *Siderastrea siderea* (Ellis and Solander, 1786) and *Montastraea faveolata* (Ellis and Solander, 1786). During the assessment period a low salinity lenses up to 9 m thick at Speyside and 3–5 m thick at the other sites, persisted around Tobago from October-December 2010 bleaching period (J. B. Alemu I, personal observation). This lens was recognised as a brown turbid layer. Salinity readings within this layer ranged between 24.7–27.0 ppt.

## Method

### Ethics Statement

Buccoo Reef Marine Park is the only marine protected area (MPA) in Trinidad and Tobago, designated in 1973, under the Marine Area Order of the Marine Areas (Preservation and Enhancement) Act 1970 and is managed by the Tobago House of Assembly (THA). There is no official licensing system to grant permission for work within the MPA; however a memorandum of understanding (MOU) exists between the Institute of Marine Affairs and the THA which permits the research conducted by the IMA within the MPA. It was against this MOU that the IMA was granted permission the bleaching assessment within the MPA in aid of management. Letters of intent were sent prior to each field visit and the field team comprised members of the THA. No permissions were required for work at Speyside and Culloden as these were not nationally protected areas. Field work did not involve contact with or removal of any endangered or protected marine species on any site. No external funding sources were required for this study.

### Data Collection

#### Ocean temperature monitoring

Ocean temperature was monitored using a combination of *in situ* meters and sea surface temperatures (SSTs) readings gathered using remote sensing techniques. *In situ* temperature data were collected near continuously at 10 m depth at all study sites from September 2009 to March 2011 using Onset HOBO Water Temp Pro loggers. Data were collected hourly, but converted to daily averages for average comparisons. Standard deviation (SD) was used to compare the variation of *in situ* temperatures among sites, as a more robust metric to mean or maximum temperature [10]. Fifty (50) km resolution night-time SSTs provided by the National Oceanographic and Atmospheric Administration Coral Reef Watch (NOAA CRW) programme [11] were used to monitor sea surface temperatures. These SSTs were recorded twice weekly, averaged and converted to monthly averages for subsequent analyses spanning the period 2001 to 2011. The bleaching threshold for corals in Tobago were considered to be 29.5°C as prescribed by the NOAA CRW; which was derived from a strong correlation between SSTs and coral bleaching.

#### Coral bleaching assessment

Coral bleaching was estimated monthly, over six successive months (October 2010 to March 2011) using the web-based standardised protocol – Bleaching Levels of the Atlantic and Gulf Reef Rapid Assessment (BLAGRRA) (http://www.agrra.org/BLAGRRA/). Four non-overlapping 10×2 m permanent belt transects were placed at the 10–12 m depth was used to assess the bleaching impact. At each site the length and width of all hard coral colonies >5 cm within each transect was measured. On all measured colonies; visual estimates of the percent bleaching and percent bleaching-induced mortality (hereafter called bleaching mortality) were conducted *in situ* by trained divers. Non-bleached colonies were assigned a score of 0% (no live tissue bleached) and bleached colonies were assigned scores between 1 and 100% reflective of the percent of live tissue that was bleached. Mortality was calculated monthly, as the change in percent bleached area to dead tissue (usually recognized as new areas of algal growth abutting bleached tissue). Only two transects were surveyed at Culloden in November 2010 and December 2010 due to hazardous environmental conditions and poor visibility, otherwise the full complement of transects was surveyed.

#### Benthic assessment

Benthic cover was sampled at three intervals: i) before the bleaching event (August/September 2010); ii) one year after the bleaching assessment (September 2011); and iii) two years after the bleaching assessment (September 2012). The September 2012 benthic assessment was not conducted at Speyside, therefore the comparison in benthic cover at Speyside was done only between 2010 and 2011. All surveys were conducted in the same area as bleaching surveys. Benthic cover was determined using non-overlapping photoquadrats along five 10×1 m, permanent transects as described by Hill and Wilkinson [12]. A total of fifty 1.0 m^2^ photos were used to assess each site using the Coral Point Count with Excel (CPCe) programme [13]. Sixty random points were overlaid onto each photoquadrat, and the benthos under each point was identified to the lowest taxonomic level, for a total of 3000 points per site. Benthic categories presented are hard coral and macroalgal cover.

### Data analysis

Overall differences in bleaching response (bleaching and mortality) among sites over the entire assessment period was tested using a repeated measured Friedman's test (n = 6) with Dunn-Bonferroni pairwise comparisons. The bleaching response of four hard coral taxa (*C. natans, S. siderea, M. cavernosa* and *M. faveolata*) was compared between and within sites. These taxa represented important reef building taxa that are commonly encountered on all the study reefs. Colonies were used as the replicates in the taxa comparisons, however this resulted an unbalanced design. To mitigate against this Bonferroni adjustments were applied to the critical value to control for Type I errors across tests, resulting in an α of 0.0125 being used. An unbalanced general linear model (GLM) with Bonferroni multiple comparisons was used for between site taxa comparisons, with site and taxa as fixed factors. The Kruskal-Wallis (K-W) test with Mann-Whitney *U* pairwise comparisons was used for within site taxa comparisons. All taxa bleaching comparisons were conducted using observations collected only at the onset of the bleaching event in October 2010. The significant of change in cover of taxa before and after bleaching was also conducted using ANOVA test with Tukey post hoc at Buccoo and Culloden. Mann-Whitney *U* test was used for pre- and post-bleaching taxa comparisons at Speyside.

## Results

### Ocean temperature monitoring

**Figure 2:**
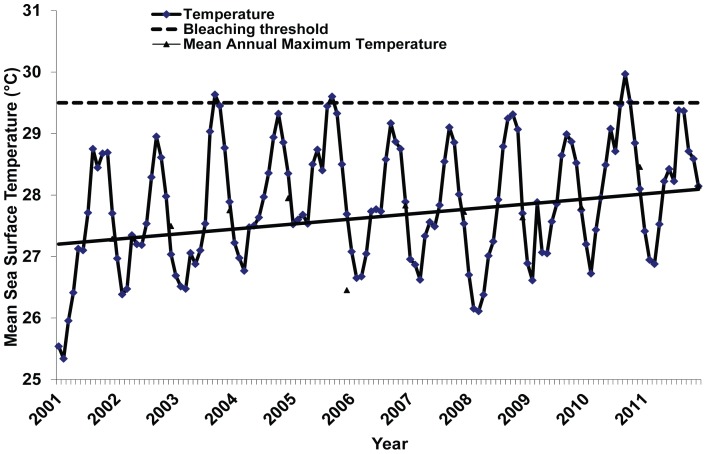
Averaged monthly sea surface temperatures (SST) for Tobago derived from the 50 km night time NOAA Coral Reef Watch Product.

**Figure 3:**
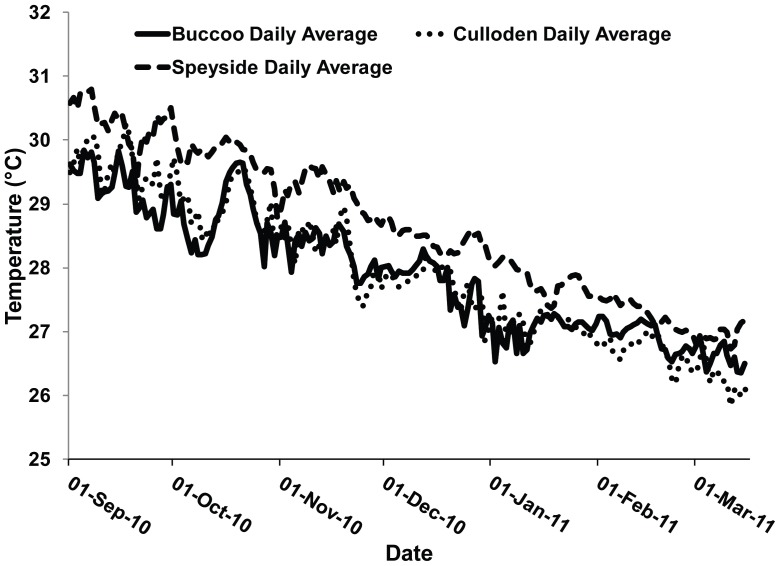
Average daily *in situ* (10 m) temperatures at Buccoo, Culloden and Speyside reefs during the 2010 mass bleaching event (September 2010 to March 2011).

Unusually warm SSTs persisted around Tobago for approximately 64 continuous days. These temperatures were the warmest recorded for Tobago over the last decade; and a decadal analysis of SST over the period 2001 to 2011 suggests that SSTs were steadily increasing (Figure 2). Generally, temperature spikes above 28.5°C coincided with mild to severe bleaching events on the island. The maximum SST recorded at the onset of the bleaching event was 30.5°C. This was almost a degree warmer than temperatures measured the previous year at the same time. Maximum *in situ* temperatures were also recorded at the onset of the bleaching event which ranged between 28.0–31.0°C among sites (Figure 3). The highest temperatures were noted at Speyside, which was as much as 0.74°C±0.02 higher than any other site (Table 1). Additionally, bleaching inducing temperatures (>29.5°C) persisted in Speyside (52 days) for a longer period than Buccoo (13 days) and Culloden (18 days).

**Table 1:** Profile of extreme *in situ* temperatures experienced at Buccoo, Culloden and Speyside over the period October 2010 to March 2011 during the 2010 mass bleaching event.

Temperature	Buccoo (days)	Culloden (days)	Speyside (days)
>30°C	0	4	20
>29.5°C	13	18	52
>29.0°C	32	44	69
Overall max Temp (°C)	30.2	30.4	31.2
Mean (°C ± SD)	27.7±0.06	27.6±0.07	28.5±0.08

### Coral bleaching assessment

Approximately 650 colonies across 30 taxa and three sites were monitored per month. Most taxa showed some bleaching, with some taxa being more susceptible than others. Coral bleaching, either as partial bleaching or complete bleaching, was observed in 22 out of 30 taxa. Taxa such as *M. faveolata*, *M. cavernosa*, *S. siderea*, *C. natans*, *D. strigosa*, *Meandrina meandrites* (Linnaeus, 1758) and *Eusmilia fastigiata* (Pallas, 1766) showed extensive bleaching (>75% mean percent bleaching per colony), with some partial bleaching mortality. Most taxa exhibited between 5–50% bleaching. In rare instances, cryptic taxa such as *Mycetophyllia ferox* (Wells, 1973) and *M. aliciae* (Wells, 1973) showed no noticeable bleaching but rather completely died within days of the onset of the bleaching event. Bleaching was not observed on *Acropora palmata* (Lamarck, 1816), *Stephanocoenia intersepta* (Lamarck, 1816), *Isophyllastraea rigida* (Dana, 1848), *Dichocoenia stokesi* (Milne Edwards, 1848), *Diploria clivosa* (Ellis & Solander, 1786), *Mussa angulosa* (Pallas, 1766), *Scolymia wellsi* (Laborel, 1967) and *Leptoseris cucullata* (Ellis & Solander, 1786); but these were rarely encountered on surveys.

**Figure 4:**
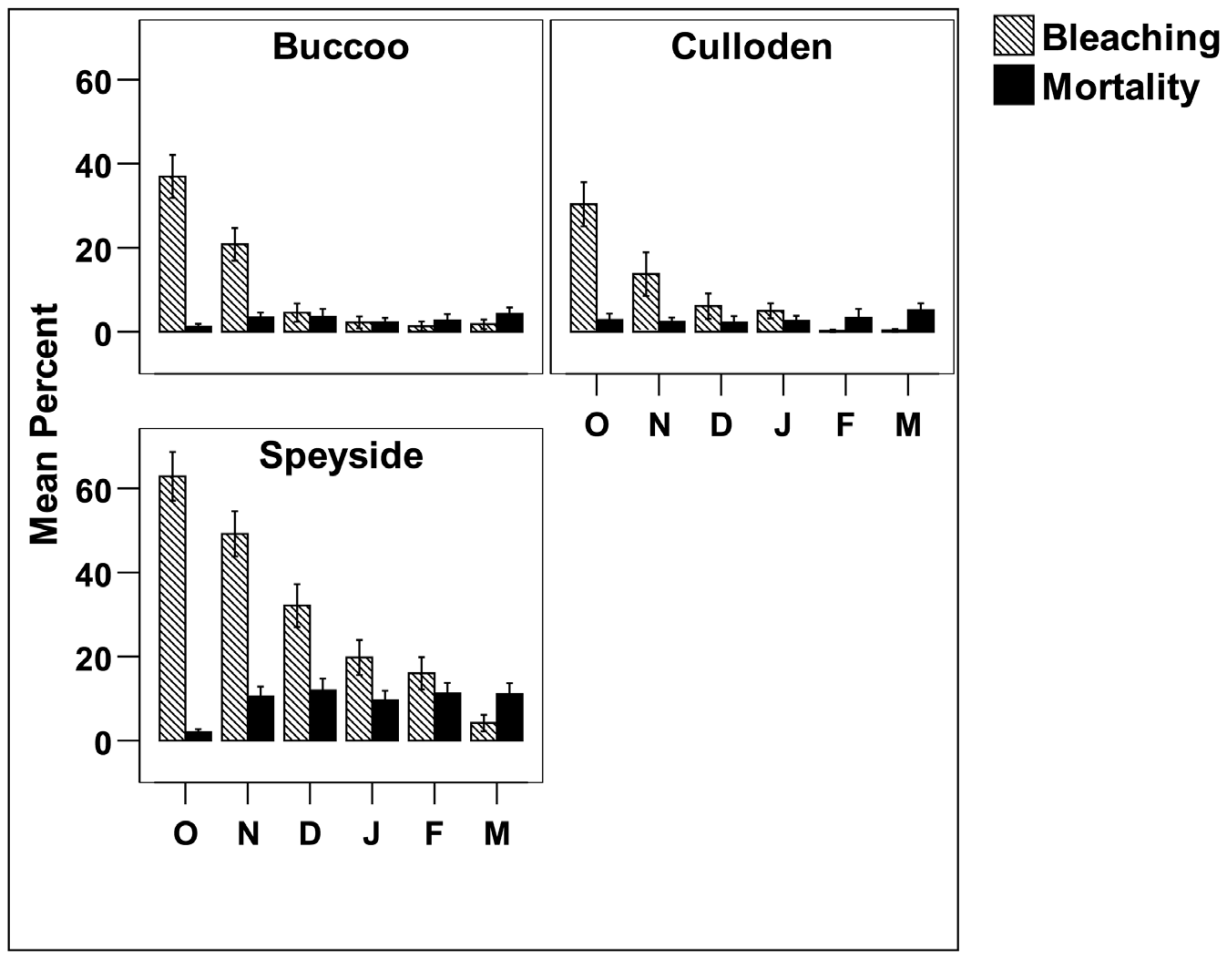
Mean percent coral bleaching and new mortality per month at Buccoo, Culloden and Speyside during the 2010 mass bleaching event (October 2010 to March 2011). Bars represent standard error. O = October 2010, N = November 2010, D = December 2010, J = January 2011, F = February 2011 and M = March 2011.

Bleaching response among sites at the onset of the bleaching event was compared using an unbalanced GLM, which gave highly significant (p<0.001) main effects of site and taxa, and site-taxa interactions (Table 2). Bonferroni pairwise comparisons revealed that bleaching response was significantly higher at Speyside; and that *M. faveolata*, *S. siderea* and *M. cavernosa* bleached significantly more than *C. natans*. The interaction significant effect were attributable to the markedly higher bleaching response of most taxa at Speyside (p<0.001) compared to the other sites, due to a stronger bleaching signal (i.e. warmer ocean temperatures). At the onset of the bleaching event Speyside exhibited 61.8%±10.0 bleaching, followed by Buccoo with 38.6%±9.3 bleaching and, lastly Culloden with 31.8%±15.2 bleaching (Figure 4).

**Table 2:** Unbalanced GLM comparing the bleaching response (bleaching and bleaching mortality) at the onset of the 2010 mass bleaching event, between three reef and four reef building taxa in Tobago.

Bleaching					
Source	Type III Sum of Squares	df	Mean Square	F	Sig.
Corrected Model	67.001^a^	11	6.091	37.97	.000
Intercept	159.930	1	159.930	996.97	.000
Taxa	20.050	3	6.683	41.66	.000
Location	18.126	2	9.063	56.50	.000
Taxa * Location	33.464	6	5.577	34.77	.000
Error	50.531	315	0.160		
Total	280.167	327			
Corrected Total	117.532	326			
Mortality					
Corrected Model	2078.842^a^	11	188.986	2.46	.006
Intercept	3620.937	1	3620.937	47.10	.000
Taxa	1427.530	3	475.843	6.19	.000
Location	261.237	2	130.618	1.70	.185
Taxa * Location	383.673	6	63.945	0.83	.546
Error	24214.871	315	76.873		
Total	30324.000	327			
Corrected Total	26293.713	326			

**Figure 5:**
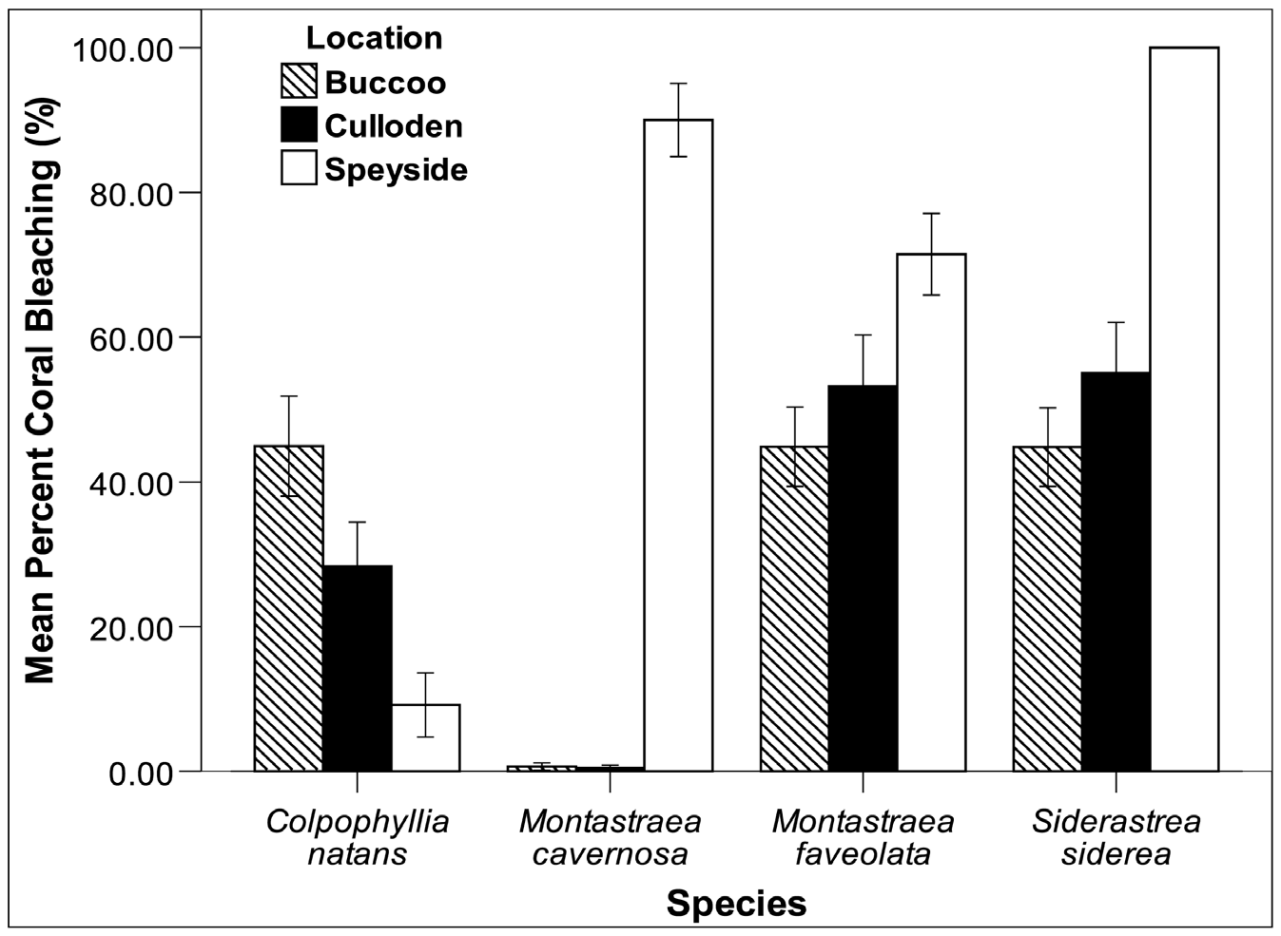
Percent bleaching of four framework building taxa at Buccoo, Culloden and Speyside following the onset of the bleaching event (October 2010). Bars represent standard error.

**Figure 6:**
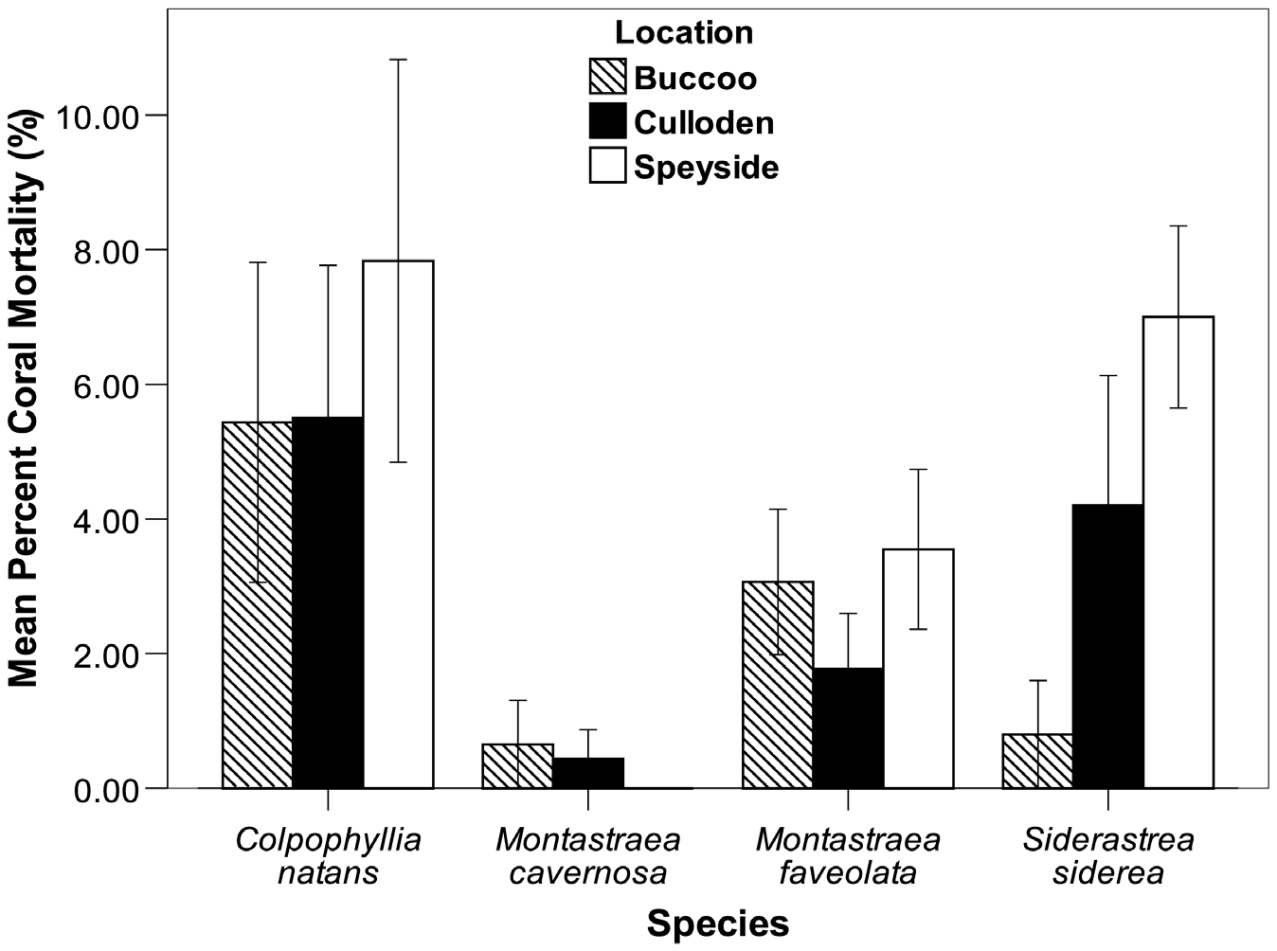
Percent bleaching mortality of four framework building taxa at Buccoo, Culloden and Speyside following the onset of the bleaching event (October 2010). Bars represent standard error.

K-W tests conducted at each site to evaluate the within site differences in bleaching among *C. natans*, *M. faveolata*, *M. cavernosa* and *S. siderea* were significant at all sites (p<0.001) (Figure 5). Follow-up pairwise tests indicated significant differences in bleaching between *C. natans* and the other three taxa observed, but there was no difference in bleaching between *M. faveolata*, *M. cavernosa* and *S. siderea* at Buccoo and Culloden. At Speyside however, significant differences in bleaching existed for most pairwise comparisons except for between *M. cavernosa* and *S. siderea*. Bleaching mortality of taxa was also compared within sites. At Buccoo and Culloden there was no significant difference in bleaching mortality among taxa (p>0.05) (Figure 6). However, at Speyside pairwise comparisons indicated that there was no difference in bleaching mortality between taxa, except for between *M. cavernosa* and *C. natans* and *M. cavernosa* and *S. siderea*, where very low *M. cavernosa* mortality was noted.

Overall, a repeated measures Friedman's test used to compare the mean bleaching among sites over a six month period, determined that bleaching differed significantly among locations (Friedman's test, χ2 = 10.18, df = 2, p<0.01). *Post hoc* tests revealed that bleaching at Speyside was significantly higher (p<0.02) than Culloden only. There was no significant difference in bleaching between Buccoo and Culloden reefs (Figure 4). It is possible that this is as a result of Speyside being exposed to a longer bleaching signal (Table 1) than the other sites, eliciting a more severe bleaching response. There was no significant difference in bleaching mortality among sites (Friedman's test, χ2 = 4.00, df = 2, p = 0.135). Overall, over the entire assessment period the mean bleaching at Speyside was 30.7%±2.2, followed by Buccoo with 11.3%±1.2 and Culloden with 9.3%±1.3. The highest bleaching mortality was noted at Speyside (8.3%±1.3), followed by Buccoo (3.2%±0.3) and Culloden (2.6%±0.4).

### Benthic assessment

Pre-bleaching (August/September 2010) coral cover was 26.3% at Culloden, followed by Buccoo with 25.2% and Speyside with 17.6%. *M. faveolata* was by far the main contributor to hard coral cover at all sites, accounting for more than 50% of hard coral cover. Most other taxa contributed to <1.0% coral cover (Table 3). Twelve months following the bleaching event (September 2011), there were noticeable declines in the hard coral cover of most taxa, with some taxa showing more declines than others. The greatest decline in coral cover was observed at Culloden Reef where reefs declined to 14.7% which represented 55.8% of pre-bleaching cover; followed by Buccoo Reef with 64.2% of pre-bleaching cover and finally Speyside Reef (68.2% of pre-bleaching cover). It should be noted that Culloden Reef exhibited the lowest overall bleaching mortality but showed the greatest post-bleaching decline in coral cover. Conversely, Speyside Reef exhibited the highest bleaching mortality but the lowest post-bleaching decline in coral cover. In 2012, a further decline in coral cover to 13.8% was noted at Culloden (52.4% of pre-bleaching coral cover); whereas Buccoo experienced a small increase in coral cover to 16.4% cover (representing 65.1% of pre-bleaching coral cover). Taxa contributing the most to the post-bleaching decline in hard coral cover were *C. natans* and *M. faveolata*. Interestingly, while overall coral cover changed drastically post bleaching, most taxa did not show a significant change in cover (Table 3).

**Table 3:** Percent change in hard coral and macroalgal cover at Buccoo, Culloden and Speyside one year and two years post 2010 mass bleaching.

	Buccoo			Culloden			Speyside	
	2010	2011	2012	2010	2011	2012	2010	2011
**Hard coral taxa**	25.19	16.17	16.38	26.28	14.65	13.77	17.55	11.97
*Acropora palmata*	0.00	0.01	0.14	0.00	0.45	0.29	-	-
*Agaricia agaricites*	0.21	0.22	0.23	0.31	0.16	0.09	0.10	0.11
*Agaricia fragilis*	<0.01	<0.01	<0.01	<0.01	<0.01	<0.01	<0.01	<0.01
*Agaricia lamarcki*	0.21	0.00	0.00	0.10	0.07	0.00	<0.01	<0.01
***Colpophyllia natans***	6.98	2.77	3.50	1.90	1.50	1.96	1.41	1.05
*Dendrogyra cylindrus*	<0.01	<0.01	<0.01	<0.01	<0.01	<0.01	-	-
*Dichocoenia stokesi*	<0.01	<0.01	<0.01	0.05	0.11	0.01	-	-
*Diploria clivosa*	<0.01	<0.01	<0.01	<0.01	<0.01	<0.01	-	-
*Diploria labyrinthiformis*	0.31	0.28	0.36	0.35	0.11	0.15	<0.01	<0.01
***Diploria strigosa***	1.13	0.85	0.73	1.80	1.77	1.97	<0.01	<0.01
*Eusmilia fastigiata*	0.32	0.21	0.05	-	-	-	0.20	<0.01
*Favia fragum*	<0.01	<0.01	<0.01	<0.01	<0.01	<0.01	<0.01	-
*Isophyllastraea rigida*	<0.01	<0.01	<0.01	<0.01	<0.01	<0.01	<0.01	<0.01
*Leptoseris cucullata*	<0.01	<0.01	<0.01	<0.01	<0.01	<0.01	<0.01	<0.01
*Madracis decactis*	0.31	0.02	0.02	0.10	0.04	0.03	0.10	0.00
*Madracis mirabilis*	<0.01	<0.01	<0.01	0.26	0.39	0.10	0.50	0.00
*Meandrina meandrites*	0.01	0.02	0.05	0.29	0.18	0.46	1.01	0.00
*Millepora alcicornis*	0.31	0.12	0.14	1.57	0.80	0.68	<0.01	<0.01
*Montastraea franksi*	<0.01	<0.01	<0.01	<0.01	<0.01	<0.01	<0.01	<0.01
*Montastraea cavernosa*	0.99	0.17	0.29	0.91	0.31	0.51	0.31	0.10
*Montastraea faveolata*	11.88	10.13	9.65	17.51^ab^	7.59^a^	6.74^b^	10.97	8.32
*Mussa angulosa*	<0.01	<0.01	<0.01	<0.01	<0.01	<0.01	<0.01	<0.01
***Mycetophyllia aliciae***	<0.01	<0.01	<0.01	0.10	0.04	0.01	<0.01	<0.01
***Mycetophyllia ferox***	0.01	0.02	0.07	0.01	0.01	0.01	<0.01	<0.01
*Porites astreoides*	0.11	0.23	0.10	0.26	0.25	0.17	0.52	0.32
*Porites furcata*	<0.01	<0.01	<0.01	<0.01	<0.01	<0.01	<0.01	<0.01
*Scolymia wellsi.*	0.02	0.02	0.02	0.01	0.05	0.02	<0.01	<0.01
*Siderastrea radians*	0.10	0.01	0.00	0.01	0.04	0.02	<0.01	<0.01
***Siderastrea siderea***	2.32	1.12	1.05	0.77	0.81	0.56	2.43	2.08
*Stephanococoenis intercepta*	<0.01	<0.01	<0.01	<0.01	<0.01	<0.01	-	-
**Macroalgae**	15.87^a^	26.65	37.90^a^	20.68^a^	41.85	54.50^a^	4.08^a^	9.57^a^

The same superscript indicate significant pairwise comparisons (p<0.05) for reef taxa (bold print).

## Discussion

Elevated temperatures, as high as 30.5°C, during the summer months of 2010 triggered a mass bleaching event in Tobago. The result was widespread paling, partial bleaching, complete bleaching or bleaching mortality on several hard coral taxa. Massive framework corals such as *Montastraea* spp., *C. natans*, *Diploria* spp., and *S. siderea*, as well as the tubular coral *E. fastigiata* and the plating *M. meandrites* were the most affected by coral bleaching, with some taxa showing as much as 100% bleaching and 80% mortality (Table S1). These taxa represent some of the major contributors to hard coral cover, and their condition might influence the whole ecosystem. Interestingly, while several taxa exhibited partial mortality, there was no significant difference in species cover between pre- and post- bleaching for most affected taxa; although framework builders have not recovered to pre-bleaching coral cover levels.

Turbid environments have been demonstrated to reduce high solar irradiance (UV) and protect corals from bleaching during periods of thermal stress [14]. The waters on the Caribbean coast of Tobago are often quite turbid, partly due to watershed run-off from the mainland and discharge from the South American continent. The result is reduced UV penetration and by extension a weaker bleaching signal [15]. However, it is more likely however, that corals on the Caribbean coast; Buccoo Reef and Culloden Reef, did not bleach extensively because they experienced lower and shorter thermal exposures. Conversely, the bleaching response on the Atlantic coast may have been enhanced by the input of a strong pulse of freshwater resting directly atop Speyside Reef.

Much like heat stress, low salinity and poor water quality disrupt the symbiotic relationship between zooxanthellae and host, resulting in or exacerbating bleaching [16]–[18]. During this study a turbid, low-salinity lens surrounded Tobago, penetrating to ∼9 m depth at Speyside, and between 3–5 m depth at the other sites. It is plausible that this lens may be associated with the discharge from the South American continent [5]. We hypothesize that at Speyside, the bleaching response could have been exacerbated as a result of the exposure of corals to nutrient rich water, which possibly lowered the thermal tolerance of corals, and by extension, exacerbated bleaching impacts [17]. Most taxa reflected the gradient of heat stress among sites. Water temperatures and bleaching response of *M. faveolata*, *S. siderea* and *M. cavernosa* were highest at Speyside, followed by Culloden and Buccoo. In contrast, of the four framework builders observed, *C. natans* bleached the least at Speyside, but the most at Buccoo. Interestingly, *M. cavernosa* showed very little bleaching at Buccoo and Culloden (<5% bleaching), but exhibited ∼90% bleaching at Speyside. Similar studies in the Caribbean have also noted the high vulnerability of several taxa such as *S. siderea* and other faviids to bleaching [19]–[21]. Recently *M. faveolata* and *C. natans* have also been recognised as highly susceptible species within the Caribbean [22], [23]. *M. cavernosa* however, has been noted to exhibit very low bleaching response [24], but the very high bleaching response at Speyside was likely to be as a result of the very high temperature and confounding environmental conditions.

Declining coral cover on reefs following the bleaching event provided an opportunity for macroalgae to overwhelm already weakened corals; further exacerbating reef stress. All sites showed twofold increases in macroalgal cover of over 130%. It should be noted though, that pre-bleaching Buccoo and Culloden showed approximately 16% and 21% macroalgal cover, whereas Speyside only exhibited ∼4% macroalgal cover. So, while there was a doubling of algal cover at sites, macroalgal cover at Speyside was still quite low. In fact, strong year round currents at Speyside make it difficult for algal settlement onto the reef substrate; by extension contributing to the prevention of algal dominated reefs [25] and improving coral reef resilience.

The spatial bleaching patterns from 2005 [7] and 2010 suggest that Atlantic sites, such as Speyside are more likely to bleach than the Caribbean reefs such as Buccoo and Culloden. The 2010 bleaching event was the most severe bleaching event in Tobago's beaching history, but while bleaching was high, mortality was low on most reefs. A stronger bleaching signal (longer exposure and higher temperatures) at Speyside made this reef even more vulnerable to bleaching impacts. In fact, most reef builders (*M. cavernosa*, *M. faveolata* and *S. siderea*) exhibited significantly (p<0.05) higher bleaching and moderate to low mortality at Speyside than at the other sites. At Buccoo and Culloden *M. cavernosa* appeared to be the most resilient taxa, where it exhibited relatively low bleaching and mortality at the onset of the bleaching event. *C. natans* however, was the most vulnerable of reef building taxa to bleaching impacts; where colonies experienced moderate to low bleaching followed by relatively high mortality across sites.

By the 2010 mass coral bleaching there was a severe reduction in hard coral cover; especially of the main framework building taxa *M. faveolata*, *S. siderea* and *C. natans* that are responsible for maintaining reef integrity. The future of reefs is tenuous at best given the projected trajectory of more frequent and severe bleaching within the Caribbean basin [26]. Of particular concern for Tobago is the impact of bleaching on the goods and services received from the reefs, and which represent a significant contributor to Tobago's annual gross domestic product (∼USD 100–160 million) [27]. The ability of reef building taxa to adapt stronger and more frequent bleaching signals, coupled with the ability of reef management to identify and conserve nodes of reef resilience is imperative to the future of Tobago's reefs. While the science of reef resilience is very much in its infancy in Tobago, the insight from this study will aid in the prioritisation of efforts towards reef or species conservation.

## Supporting Information

Table S1 **Total bleaching and mortality estimates of four reef building taxa during the onset of the 2010 mass bleaching event.**
(DOCX)
