# Correction: Mass coral bleaching in 2010 in the Southern Caribbean

**DOI:** 10.1371/journal.pone.0092542

**Published:** 2014-03-24

**Authors:** The PLOS ONE Staff

Errors were introduced to Table 3 during the production process. *Mycetophyllia aliciae* and *Mycetophyll ferox* are bolded in error. ***Montastraea faveolata*** and ***Montastraea cavernosa*** are not bolded in error. The corrected version of Table 3 can be viewed here. The publisher apologizes for these errors.

**Table 3:** Percent change in hard coral and macroalgal cover at Buccoo, Culloden and Speyside one year and two years post 2010 mass bleaching.

	Buccoo			Culloden			Speyside	
	2010	2011	2012	2010	2011	2012	2010	2011
**Hard coral taxa**	25.19	16.17	16.38	26.28	14.65	13.77	17.55	11.97
*Acropora palmata*	0.00	0.01	0.14	0.00	0.45	0.29	-	-
*Agaricia agaricites*	0.21	0.22	0.23	0.31	0.16	0.09	0.10	0.11
*Agaricia fragilis*	<0.01	<0.01	<0.01	<0.01	<0.01	<0.01	<0.01	<0.01
*Agaricia lamarcki*	0.21	0.00	0.00	0.10	0.07	0.00	<0.01	<0.01
***Colpophyllia natans***	6.98	2.77	3.50	1.90	1.50	1.96	1.41	1.05
*Dendrogyra cylindrus*	<0.01	<0.01	<0.01	<0.01	<0.01	<0.01	-	-
*Dichocoenia stokesi*	<0.01	<0.01	<0.01	0.05	0.11	0.01	-	-
*Diploria clivosa*	<0.01	<0.01	<0.01	<0.01	<0.01	<0.01	-	-
*Diploria labyrinthiformis*	0.31	0.28	0.36	0.35	0.11	0.15	<0.01	<0.01
***Diploria strigosa***	1.13	0.85	0.73	1.80	1.77	1.97	<0.01	<0.01
*Eusmilia fastigiata*	0.32	0.21	0.05	-	-	-	0.20	<0.01
*Favia fragum*	<0.01	<0.01	<0.01	<0.01	<0.01	<0.01	<0.01	-
*Isophyllastraea rigida*	<0.01	<0.01	<0.01	<0.01	<0.01	<0.01	<0.01	<0.01
*Leptoseris cucullata*	<0.01	<0.01	<0.01	<0.01	<0.01	<0.01	<0.01	<0.01
*Madracis decactis*	0.31	0.02	0.02	0.10	0.04	0.03	0.10	0.00
*Madracis mirabilis*	<0.01	<0.01	<0.01	0.26	0.39	0.10	0.50	0.00
*Meandrina meandrites*	0.01	0.02	0.05	0.29	0.18	0.46	1.01	0.00
*Millepora alcicornis*	0.31	0.12	0.14	1.57	0.80	0.68	<0.01	<0.01
*Montastraea franksi*	<0.01	<0.01	<0.01	<0.01	<0.01	<0.01	<0.01	<0.01
***Montastraea cavernosa***	0.99	0.17	0.29	0.91	0.31	0.51	0.31	0.10
***Montastraea faveolata***	11.88	10.13	9.65	17.51^ab^	7.59^a^	6.74^b^	10.97	8.32
*Mussa angulosa*	<0.01	<0.01	<0.01	<0.01	<0.01	<0.01	<0.01	<0.01
*Mycetophyllia aliciae*	<0.01	<0.01	<0.01	0.10	0.04	0.01	<0.01	<0.01
*Mycetophyllia ferox*	0.01	0.02	0.07	0.01	0.01	0.01	<0.01	<0.01
*Porites astreoides*	0.11	0.23	0.10	0.26	0.25	0.17	0.52	0.32
*Porites furcata*	<0.01	<0.01	<0.01	<0.01	<0.01	<0.01	<0.01	<0.01
*Scolymia wellsi.*	0.02	0.02	0.02	0.01	0.05	0.02	<0.01	<0.01
*Siderastrea radians*	0.10	0.01	0.00	0.01	0.04	0.02	<0.01	<0.01
***Siderastrea siderea***	2.32	1.12	1.05	0.77	0.81	0.56	2.43	2.08
*Stephanococoenis intercepta*	<0.01	<0.01	<0.01	<0.01	<0.01	<0.01	-	-
**Macroalgae**	15.87^a^	26.65	37.90^a^	20.68^a^	41.85	54.50^a^	4.08^a^	9.57^a^

The same superscript indicate significant pairwise comparisons (p < 0.05) for reef taxa (bold print).

## References

[1] Alemu I JB, Clement Y (2014) Mass Coral Bleaching in 2010 in the Southern Caribbean. PLoS ONE 9(1): e83829 doi:10.1371/journal.pone.0083829 24400078 10.1371/journal.pone.0083829 PMC3882216

