# Supplementary material for: Mass Coral Bleaching in 2010 in the Southern Caribbean
Source: PLoS One. 2014 Jan 6;9(1):e83829. doi: 10.1371/journal.pone.0083829 (PMC3882216; doi:10.1371/journal.pone.0083829)
Supplement: Table S1 — Total bleaching and mortality estimates of four reef building taxa during the onset of the 2010 mass bleaching event. (DOCX) [file pone.0083829.s001.docx]

Table S1: Total bleaching and mortality estimates of four reef building taxa during the onset of the 2010 mass bleaching event

|  | **Bleaching** | | | **Bleaching Mortality** | | | |  |
| --- | --- | --- | --- | --- | --- | --- | --- | --- |
| **Taxa** | **Buccoo** | **Culloden** | **Speyside** | | **Buccoo** | **Culloden** | **Speyside** | |
| *Colpophyllia natans* | 100 | 90 | 100 | | 0 | 0 | 10 | |
| *Colpophyllia natans* | 100 | 0 | 75 | | 0 | 5 | 10 | |
| *Colpophyllia natans* | 100 | 0 | 0 | | 0 | 0 | 0 | |
| *Colpophyllia natans* | 90 | 70 | 0 | | 0 | 0 | 0 | |
| *Colpophyllia natans* | 90 | 60 | 0 | | 0 | 0 | 0 | |
| *Colpophyllia natans* | 60 | 50 | 0 | | 0 | 0 | 30 | |
| *Colpophyllia natans* | 50 | 40 | 0 | | 0 | 0 | 0 | |
| *Colpophyllia natans* | 45 | 40 | 0 | | 0 | 20 | 0 | |
| *Colpophyllia natans* | 45 | 20 | 0 | | 0 | 0 | 0 | |
| *Colpophyllia natans* | 20 | 20 | 50 | | 0 | 0 | 0 | |
| *Colpophyllia natans* | 15 | 15 | 40 | | 0 | 0 | 0 | |
| *Colpophyllia natans* | 8 | 100 | 0 | | 0 | 25 | 0 | |
| *Colpophyllia natans* | 5 | 90 | 0 | | 0 | 0 | 25 | |
| *Colpophyllia natans* | 0 | 90 | 0 | | 0 | 0 | 10 | |
| *Colpophyllia natans* | 100 | 45 | 0 | | 0 | 0 | 0 | |
| *Colpophyllia natans* | 100 | 0 | 0 | | 0 | 0 | 0 | |
| *Colpophyllia natans* | 80 | 0 | 0 | | 40 | 0 | 0 | |
| *Colpophyllia natans* | 80 | 0 | 0 | | 0 | 50 | 20 | |
| *Colpophyllia natans* | 75 | 0 | 0 | | 40 | 0 | 0 | |
| *Colpophyllia natans* | 60 | 0 | 0 | | 0 | 0 | 5 | |
| *Colpophyllia natans* | 40 | 0 | 0 | | 10 | 0 | 0 | |
| *Colpophyllia natans* | 20 | 50 | 0 | | 0 | 10 | 0 | |
| *Colpophyllia natans* | 20 | 0 | 0 | | 5 | 40 | 5 | |
| *Colpophyllia natans* | 20 | 30 | 0 | | 43 | 0 | 80 | |
| *Colpophyllia natans* | 15 | 0 | 0 | | 0 | 0 | 30 | |
| *Colpophyllia natans* | 10 | 0 | 5 | | 25 | 0 | 0 | |
| *Colpophyllia natans* | 0 | 0 | 5 | | 0 | 0 | 5 | |
| *Colpophyllia natans* | 0 | 0 | 0 | | 0 | 0 | 5 | |
| *Colpophyllia natans* | 0 | 40 | 0 | | 0 | 5 | 0 | |
| *Colpophyllia natans* | 0 | 0 | 0 | | 0 | 10 | 0 | |
| *Montastraea cavernosa* | 10 | 0 | 100 | | 0 | 0 | 0 | |
| *Montastraea cavernosa* | 5 | 0 | 100 | | 0 | 0 | 0 | |
| *Montastraea cavernosa* | 0 | 0 | 100 | | 0 | 0 | 0 | |
| *Montastraea cavernosa* | 0 | 0 | 100 | | 0 | 0 | 0 | |
| *Montastraea cavernosa* | 0 | 10 | 100 | | 0 | 10 | 0 | |
| *Montastraea cavernosa* | 0 | 0 | 100 | | 0 | 0 | 0 | |
| *Montastraea cavernosa* | 0 | 0 | 100 | | 0 | 0 | 0 | |
| *Montastraea cavernosa* | 0 | 0 | 100 | | 0 | 0 | 0 | |
| *Montastraea cavernosa* | 0 | 0 | 100 | | 0 | 0 | 0 | |
| *Montastraea cavernosa* | 0 | 0 | 100 | | 0 | 0 | 0 | |
| *Montastraea cavernosa* | 0 | 0 | 100 | | 0 | 0 | 0 | |
| *Montastraea cavernosa* | 0 | 0 | 100 | | 0 | 0 | 0 | |
| *Montastraea cavernosa* | 0 | 0 | 100 | | 0 | 0 | 0 | |
| *Montastraea cavernosa* | 0 | 0 | 100 | | 0 | 0 | 0 | |
| *Montastraea cavernosa* | 0 | 0 | 100 | | 0 | 0 | 0 | |
| *Montastraea cavernosa* | 0 | 0 | 100 | | 0 | 0 | 0 | |
| *Montastraea cavernosa* | 0 | 0 | 100 | | 0 | 0 | 0 | |
| *Montastraea cavernosa* | 0 | 0 | 100 | | 15 | 0 | 0 | |
| *Montastraea cavernosa* | 0 | 0 | 85 | | 0 | 0 | 0 | |
| *Montastraea cavernosa* | 0 | 0 | 80 | | 0 | 0 | 0 | |
| *Montastraea cavernosa* | 0 | 0 | 70 | | 0 | 0 | 0 | |
| *Montastraea cavernosa* | 0 | 0 | 20 | | 0 | 0 | 0 | |
| *Montastraea cavernosa* | 0 | 0 | 15 | | 0 | 0 | 0 | |
| *Montastraea faveolata* | 100 | 100 | 100 | | 5 | 0 | 30 | |
| *Montastraea faveolata* | 100 | 100 | 100 | | 5 | 0 | 15 | |
| *Montastraea faveolata* | 100 | 100 | 100 | | 0 | 0 | 10 | |
| *Montastraea faveolata* | 80 | 100 | 95 | | 0 | 0 | 10 | |
| *Montastraea faveolata* | 80 | 100 | 95 | | 0 | 0 | 10 | |
| *Montastraea faveolata* | 80 | 95 | 90 | | 0 | 0 | 5 | |
| *Montastraea faveolata* | 75 | 90 | 90 | | 0 | 0 | 5 | |
| *Montastraea faveolata* | 75 | 90 | 90 | | 0 | 10 | 5 | |
| *Montastraea faveolata* | 65 | 80 | 90 | | 0 | 5 | 0 | |
| *Montastraea faveolata* | 65 | 80 | 90 | | 0 | 0 | 0 | |
| *Montastraea faveolata* | 60 | 80 | 90 | | 0 | 0 | 0 | |
| *Montastraea faveolata* | 55 | 75 | 90 | | 0 | 0 | 0 | |
| *Montastraea faveolata* | 50 | 75 | 90 | | 20 | 0 | 0 | |
| *Montastraea faveolata* | 50 | 75 | 90 | | 10 | 0 | 0 | |
| *Montastraea faveolata* | 50 | 70 | 90 | | 0 | 10 | 0 | |
| *Montastraea faveolata* | 40 | 70 | 80 | | 0 | 0 | 0 | |
| *Montastraea faveolata* | 30 | 70 | 80 | | 20 | 0 | 0 | |
| *Montastraea faveolata* | 30 | 60 | 80 | | 10 | 0 | 0 | |
| *Montastraea faveolata* | 30 | 50 | 80 | | 0 | 0 | 0 | |
| *Montastraea faveolata* | 25 | 30 | 75 | | 0 | 0 | 0 | |
| *Montastraea faveolata* | 20 | 20 | 70 | | 10 | 0 | 0 | |
| *Montastraea faveolata* | 20 | 20 | 70 | | 0 | 0 | 0 | |
| *Montastraea faveolata* | 20 | 20 | 70 | | 0 | 0 | 0 | |
| *Montastraea faveolata* | 20 | 0 | 70 | | 0 | 0 | 0 | |
| *Montastraea faveolata* | 15 | 0 | 70 | | 0 | 0 | 10 | |
| *Montastraea faveolata* | 10 | 0 | 50 | | 15 | 0 | 0 | |
| *Montastraea faveolata* | 10 | 0 | 20 | | 0 | 10 | 10 | |
| *Montastraea faveolata* | 10 | 0 | 10 | | 0 | 0 | 0 | |
| *Montastraea faveolata* | 10 | 0 | 0 | | 0 | 0 | 0 | |
| *Montastraea faveolata* | 10 | 0 | 0 | | 0 | 20 | 0 | |
| *Montastraea faveolata* | 5 | 0 | 0 | | 0 | 0 | 0 | |
| *Siderastrea siderea* | 80 | 80 | 100 | | 0 | 20 | 15 | |
| *Siderastrea siderea* | 80 | 5 | 100 | | 0 | 5 | 20 | |
| *Siderastrea siderea* | 75 | 100 | 100 | | 0 | 0 | 15 | |
| *Siderastrea siderea* | 75 | 100 | 100 | | 0 | 0 | 15 | |
| *Siderastrea siderea* | 70 | 100 | 100 | | 0 | 0 | 15 | |
| *Siderastrea siderea* | 70 | 90 | 100 | | 0 | 0 | 15 | |
| *Siderastrea siderea* | 70 | 90 | 100 | | 0 | 0 | 10 | |
| *Siderastrea siderea* | 65 | 90 | 100 | | 0 | 5 | 10 | |
| *Siderastrea siderea* | 60 | 85 | 100 | | 0 | 15 | 10 | |
| *Siderastrea siderea* | 60 | 80 | 100 | | 0 | 0 | 10 | |
| *Siderastrea siderea* | 60 | 70 | 100 | | 0 | 0 | 10 | |
| *Siderastrea siderea* | 60 | 70 | 100 | | 0 | 0 | 10 | |
| *Siderastrea siderea* | 50 | 70 | 100 | | 0 | 0 | 10 | |
| *Siderastrea siderea* | 45 | 70 | 100 | | 0 | 0 | 10 | |
| *Siderastrea siderea* | 40 | 60 | 100 | | 0 | 0 | 0 | |
| *Siderastrea siderea* | 40 | 50 | 100 | | 0 | 0 | 0 | |
| *Siderastrea siderea* | 35 | 45 | 100 | | 0 | 0 | 0 | |
| *Siderastrea siderea* | 25 | 40 | 100 | | 0 | 0 | 0 | |
| *Siderastrea siderea* | 20 | 30 | 100 | | 0 | 0 | 0 | |
| *Siderastrea siderea* | 20 | 20 | 100 | | 0 | 0 | 0 | |
| *Siderastrea siderea* | 10 | 20 | 100 | | 20 | 20 | 0 | |
| *Siderastrea siderea* | 5 | 10 | 100 | | 0 | 0 | 0 | |
| *Siderastrea siderea* | 5 | 0 | 100 | | 0 | 0 | 0 | |
| *Siderastrea siderea* | 0 | 0 | 100 | | 0 | 0 | 0 | |
| *Siderastrea siderea* | 0 | 0 | 100 | | 0 | 40 | 0 | |
